# Analyzing the spatiotemporal pattern of the decoupling degree between carbon metabolism and economic development in village and town units

**DOI:** 10.1371/journal.pone.0296787

**Published:** 2024-04-18

**Authors:** Yanghua Zhang, Bin Liu, Hu Zhao, Weipeng Lin, Liang Cheng, Xiaogang Wang

**Affiliations:** 1 School of Architecture and Urban Planning, Shandong Jianzhu University, Jinan, China; 2 Shandong High Speed Information Group Co., Ltd. Jinan, China; Huazhong University of Science and Technology, CHINA

## Abstract

In the context of green and sustainable development and rural revitalization, analysis of the relationship between economic development and the evolution of carbon metabolism is of great significance for China’s future transformation of development models. This study analyzed the spatial characteristics and spatiotemporal evolution pattern of the decoupling status between carbon metabolism and economic development of Laiwu during two periods from 2001 to 2018 at the village and town unit scales by using the Tapio decoupling model. The results showed that the growth rate of carbon metabolism from 2001 to 2009 was significantly higher than that from 2009 to 2018. The spatial heterogeneity of the decoupling states between economic development and carbon metabolism from 2009 to 2018 was significantly stronger than that from 2001 to 2009 in two units. From 2001 to 2018, the development trend gradually trended towards spatial imbalance. The decoupling status between villages and towns had a high degree of consistency from 2001 to 2009 and inconsistency from 2009 to 2018. From 2001 to 2009, the decoupling status of about 78% of villages was consistent with that of towns. Moreover, from 2009 to 2018, the consistency reduced to 32.2%, and the decoupling status of about 48% of villages was weaker than that of towns. According to the reclassification results of different decoupling state change types, from 2001 to 2018, about 52.2% of the villages had a decoupling state evolution type of eco-deteriorated economic development, which is an unsatisfactory development trend in a short time. Moreover, about 12.1% of the villages had a decoupling state evolution type of eco-improved economic development, which is a satisfactory development trend.

## 1. Introduction

In recent years, climate warming and frequent extreme climate events have made carbon emission reduction an important issue [1,2]. At the Leaders’ Summit on Climate 2021, 40 countries and organizations announced ambitious updated climate change mitigation targets and discussed innovative pathways to a net-zero economy [3]. In 2020, China’s carbon emissions were about 10.3 billion tons, and about 7.4 tons per capita [4]. Responsible for 32% of global carbon emissions, China’s actions in carbon mitigation are vital to achieving the goal of global environmental improvement. In order to fulfill responsibilities, China has pledged to peak its emissions before 2030 and achieve carbon neutrality before 2060 [5]. In fact, China has been investing in this field through policy support and system renovation for many years [6,7]. Meanwhile, as a developing country, China needs to continue to develop its economy. In light of the correlation between carbon emission growth and economic growth, it is significant to analyze the decoupling degree between carbon metabolism and economic development. Moreover, the spatiotemporal pattern analysis of the decoupling degree is meaningful for the government to balance regional low-carbon economic development.

Previous studies have shown that a region’s carbon emission growth is closely coupled with its economic growth [8–11]. In addition, the evolutionary relationship generally conforms to the environmental Kuznets curve (EKC), which presents an inverted U shape [10,12,13]. To verify the EKC evolution trend and to analyze the causes of non-synchronous changes in the economy and carbon emissions, many evolutionary relationship studies of two elements, including carbon emissions, energy consumption, the urbanization process, and economic development, have been carried out [10]. Li examined the decoupling degree between economic growth and the production, consumption, and income-based emissions based on provincial data of China during 2012–2017, and found that more than 70% of provinces have achieved decoupling of GDP from three types of emissions [3]. To verify whether China’s development conforms to the EKC, Riti analyzed the nexus of CO_2_ emissions, economic growth, and energy consumption using different methods, and found that the turning point of China was approximately consistent with the EKC [10]. Xu investigated the decoupling state of multisectoral carbon emissions of Guangdong province from 2002–2017 and found that the electricity production and construction sectors were the largest direct and embodied carbon emission sources, and the consumption structure, consumption per capita, and population effect promoted the embodied emissions during 2002–2012 [11]. In these studies, the Tapio decoupling analysis model was a common method.

The evaluation of carbon metabolism was the vital part of these studies. Research on carbon metabolism accounting can be classified from various perspectives. From the perspective of whether to consider the spatial feature of carbon metabolism, studies can be divided into spatial [14,15] and non-spatial [16,17] accounting studies. From the perspective of the degree of refinement of research, studies can be divided into macro [6,18] and micro [11,17] carbon metabolism accounting studies. These perspectives are not independent of each other, and have a certain intersection. For the macroscopic studies, whether or not the spatial characteristics of carbon metabolism are considered, the IPCC emission coefficients-based carbon metabolism accounting method is often used at the provincial, regional, or national level [4,19–21]. This kind of study relies heavily on historical energy consumption data that are usually restricted by administrative units. For microscopic studies, spatial and non-spatial carbon metabolism analyses have some differences in the carbon accounting method. The former usually uses the spot investigation-based modeling and remote sensing-based land classification method to extract spatial carbon emissions [14,15,22]. The spot investigation-based modeling method requires some accurately measured points to support and test the space interpolation model, thus, it can be prohibitively expensive and time-consuming [22]. The remote sensing-based land classification method estimates the carbon metabolism by the carbon emission and sequestration coefficients of each land use and land cover (LULC) type extracted by remote sensing-based classification 14,15]. This method has many advantages at multiple spatial scales and a long time series analysis of carbon metabolism due to the large reserve of remote sensing data, especially the Landsat images [23]. Moreover, some progress has been made in the investigation of carbon emission and sequestration coefficients of different land types [14]. The latter usually focuses on the carbon metabolism between different sectors or industries through input-output, life cycle assessment, and material flow analysis methods [16,17,24,25].

The spatial analysis of carbon metabolism is helpful for urban greenhouse effect control and balanced improvement of different regional environments [26,27]. As previously mentioned, the spatial carbon metabolism accounting methods mainly contain spot investigation-based modeling and remote sensing-based LULC classification methods. The remote sensing-based LULC classification method is widely used due to the wide-area and long-time coverage of the remote sensing data [14,26]. According to previous studies, vegetation and water bodies have carbon net sequestration effects and cultivated land has a carbon net emission effect [14,28,29]. Construction land, especially industrial land, has high-intensity carbon net emission effects due to the high consumption of building materials and fossil energy. Therefore, some studies have concluded that urbanization significantly promotes carbon emissions [14,15]. Zhang studied the spatial pattern of Beijing’s carbon metabolism over 5 years through the LULC classification method and found that the carbon emission area was consistent with the built-up area, and the rate of carbon emission was significantly faster than that of absorption across these years [14]. Xia studied the impact of urban expansion on carbon emissions in Beijing from 1990 to 2010 and found that the urban expansion obviously broadened the area of carbon emissions and gave rise to carbon emissions in the urban center [15]. Chen studied the spatial heterogeneity of the decoupling degree between carbon metabolism and economic development of Guangdong province from 2005–2015 based on the urban panel data, and found that in 2005–2010, most cities were in a weak decoupling state, and in 2010–2015, some cities had changed to a strong decoupling state [19].

Some studies have focused on the decoupling relationship between economy and the environment [3,10,30]. Moreover, spatial heterogeneity analysis of the decoupling degree has been carried out at national, provincial, municipal, and county levels based on statistical data [3,11,31]. However, few studies have focused on decoupling analysis of economic development and carbon metabolism at the level of villages and towns, especially research on the consistency of the decoupling degree at these levels.

The development of villages and towns is a key focus area of China’s government. For this reason, China has put forward a major development strategy for rural revitalization [32,33]. However, the economic development of villages and towns at the expense of environmental quality is no longer permitted. Therefore, research on the decoupling degree between carbon metabolism and economic development at the village and town unit level is of great significance to support rural revitalization and green development simultaneously. The spatiotemporal pattern analysis of that decoupling degree is very important for balanced development of the regional environment and economy.

This study selected LaiWu city as an example to investigate the following topics: changes in the spatial pattern of the decoupling degree between carbon metabolism and economic development at the village and town unit level, and inconsistency of the decoupling degree between the village and town unit level. To do so, the structure of this manuscript has been organized as follows: first, the carbon metabolism of city from 2001 to 2018 was identified based on time series LULC data. Next, the spatiotemporal pattern of the decoupling degree between carbon metabolism and economic development was analyzed at the village and town unit level separately. Finally, the integrated analysis of the decoupling degree at the village and town unit level was undertaken to extract the inconsistency of development at different administrative levels.

## 2. Study area and data

### 2.1 Study area

**Fig 1:**
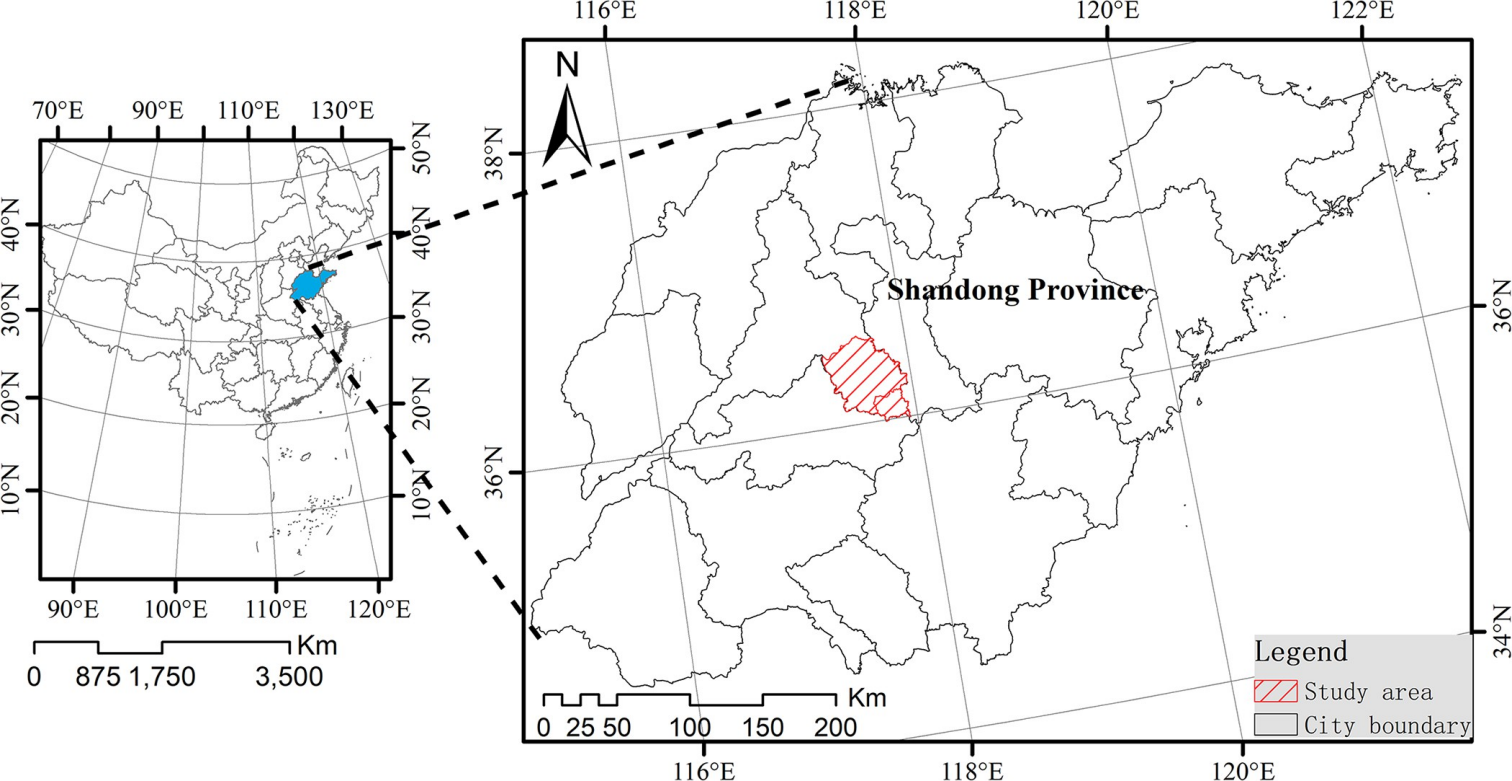
The spatial location and scope of the study area.

Laiwu City was selected as the study area. It is located in the middle of Shandong Province, China, at the eastern foot of Mount Tai (36°02´ N–36°33´ N, 117°19´E–117°58´ E; Fig 1). The topography of the study area is relatively complex, with the region being rich in vegetation. Mountains and hills account for approximately 85% of the study area, whereas plains account for only approximately 11%. Laiwu has abundant coal resources and is an important coal base in Shandong Province. Owing to its plentiful resources, Laiwu has been a steel production base in Shandong for several years. However, these activities have caused extensive damage to the environment. Since 2012, the Chinese government has continuously strengthened awareness of environmental protections and high-quality development [34]. Laiwu has also gradually eliminated the resource-dependent development model. Thus, this area has experienced a rough development phase with the loss of considerable natural ecological resources; however, it is possible that Laiwu will experience high-quality development that will improve the area’s economy and ecology simultaneously. Therefore, Laiwu was taken as the research object in this study.

### 2.2 Data

**Fig 2:**
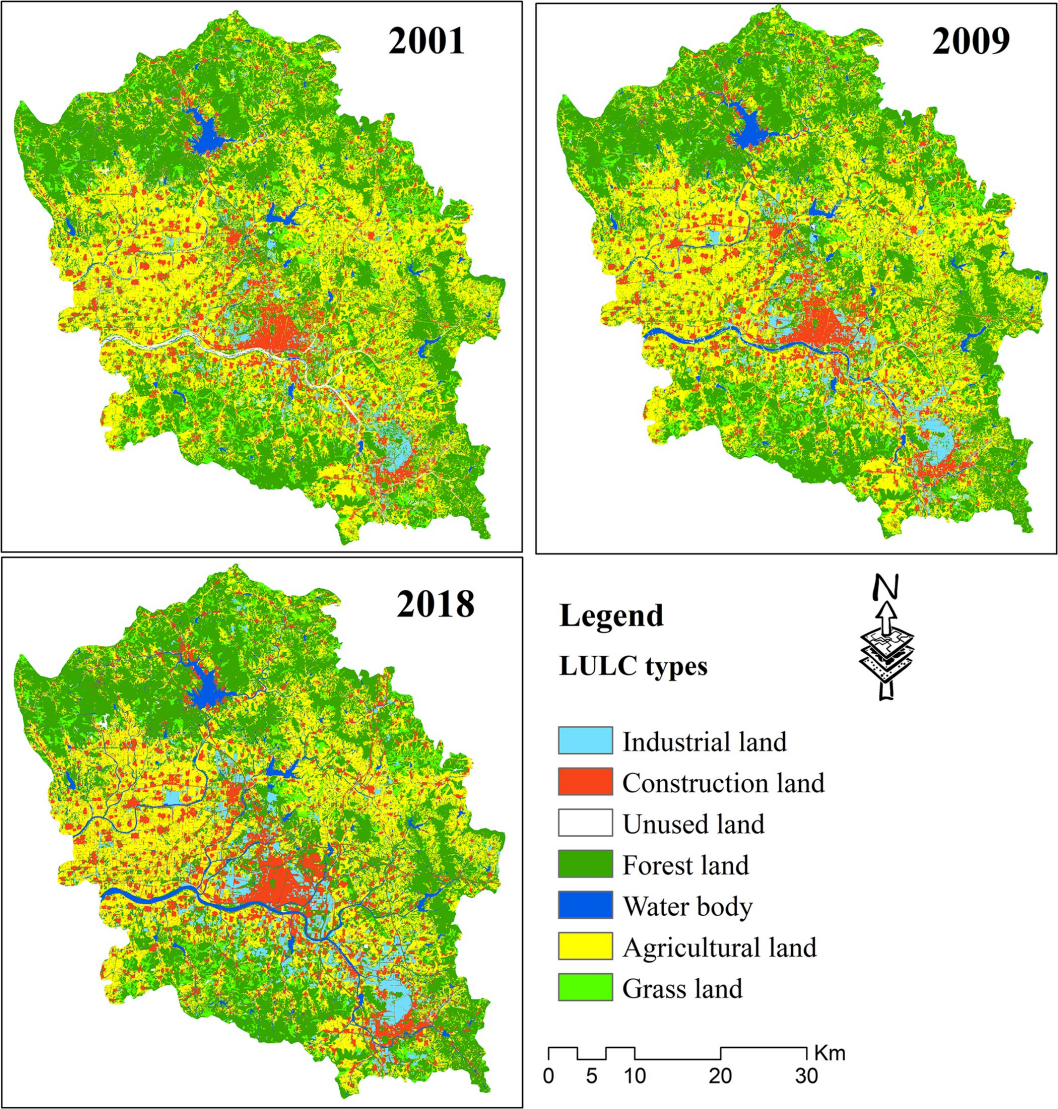
The land use and land cover classification maps for 3 years.

The data used in this study are shown in Table 1, which shows data related to time series LULC, point of interest (POI), land surveys, and GDP, as well as the vector administrative data of villages and towns. Multitemporal LULC data were extracted based on Landsat images for 2001, 2009, and 2018 with a resolution of 30 × 30 m. These data were downloaded from https://earthexplorer.usgs.gov/, with path 122 row 35. The support vector machine method [35] was used in the initial LULC classification process. To ensure classification accuracy, POIs and land survey data were used as auxiliary data to refine the initial classification data, and the error matrix [36] was used to evaluate the accuracy of the final LULC classification results. The LULC types mainly included industrial land, forest land, construction land, agriculture land, water bodies, grassland, and unused land (Fig 2). The overall accuracies of LULC classification maps were all greater than 90%, and the Kappa coefficients were all higher than 0.87.

**Table 1:** Data used in this study.

Category	Data	Year(s)	Resolution	Data source
Land use and land cover	Land use and land cover data	2001, 2009, 2018	30 m	Landsat images downloaded from the USGS website (https://earthexplorer.usgs.gov/)
Point of interest (POI)	POIs of entertainment facilities, industrial sites, schools, parking spots, public service facilities, business services, administrative agencies	2005, 2018		Baidu online map
Vector data	Administrative boundary	2017		Bureau of Planning and Natural Resources
Economy data	Gross domestic product (GDP)	2000, 2010, 2019	1 km	Resource and Environment Science and Data Center website (https://www.resdc.cn/)
Energy data	Raw coal, washed coal, coke, crude oil, fuel oil, gasoline, liquefied petroleum gas, natural gas, etc.	2001, 2009, 2018		China Energy Statistical Yearbook, Laiwu Statistical Yearbook

The time series GDP data and administrative boundary maps were downloaded from the website of the Resource and Environment Science and Data Center (https://www.resdc.cn/). These GDP spatial distribution data were created considering multiple factors closely related to human economic activities, such as land use type, nighttime light intensity, and residential density [37,38], and have been widely used in studies [39,40]. The spatial resolution of GDP data was 1 × 1 km, and the unit was 10,000 yuan/km^2^. Moreover, in order to mitigate the impact of inflation on GDP, the GDP for 3 years was corrected by the consumer price index [41,42].

## 3. Methods

### 3.1 Accounting for carbon metabolism

The carbon metabolisms from 2001 to 2018 of the study area were determined based on time series LULC data, which were extracted from Landsat images (Fig 2). Carbon metabolism includes emission and sequestration [15]. Different LULC types have different carbon metabolism intensities. Currently, land use carbon metabolism accounting generally separates construction land from non-construction land [15,26]. Calculation of the carbon metabolism of non-construction lands generally directly uses the carbon metabolism coefficients and areas of each LULC [14]. Moreover, calculation of the carbon metabolism of construction lands generally indirectly uses the energy consumption statistical data and carbon metabolism coefficients of energies [15]. In addition, Zhou innovatively used multisector energy consumption statistics to correct the carbon metabolism coefficients of LULC types, then used the corrected coefficients in the carbon metabolism calculation process [43]. Chen established a micro-mechanism model of a blast furnace iron-making system based on the network of industrial metabolism via analyzing the mass energy balances and simulating the industrial metabolic pathways instead of using the emission coefficients [16]. Due to the difficulty in obtaining detailed energy consumption statistics about non-construction land, this study used the carbon metabolism coefficients method for non-construction lands, and the energy consumption statistics-based method for construction lands.

According to previous studies, the main carbon emission land includes industrial, construction, and agriculture lands [19]. Moreover, the main carbon sequestration land includes forest land, water bodies, and grassland [44,45]. The carbon metabolisms of forest land, agriculture land, water bodies, grassland, and unused land were mainly determined by the empirical carbon metabolism coefficient [28,45]. The calculation formula is as follows:

$$C_{i}=k_{i}S_{i}$$

where *C*_*i*_ is the carbon metabolism quantity of the LULC type *i*. The *S*_*i*_ is the area of the LULC type *i*. The *k*_*i*_ is the carbon metabolism coefficient, which represents the carbon metabolism quantity per unit area throughout the year of the LULC type *i*. A negative value of *k*_*i*_ represents the net carbon sequestration of type *i*, and a positive value of *k*_*i*_ represents the net carbon emission of type *i*. The k coefficient was −0.0657 *kgCm*^−2^*yr*^−1^ for forest land, −0.0021 *kgCm*^−2^*yr*^−1^ for grassland, −0.0303 *kgCm*^−2^*yr*^−1^ for water bodies, −0.00005 *kgCm*^−2^*yr*^−1^ for unused land, and 0.0422 *kgCm*^−2^*yr*^−1^ for agriculture land.

The carbon metabolisms of industrial and construction lands mainly come from production and living, transportation, and other energy consumption processes. The energy balance table algorithm was used in this study [46]. The selected energy sources included raw coal, refinery dry gas, cleaned coal, crude oil, briquette, liquefied petroleum gas, kerosene, coke oven gas, diesel, coke, gasoline, fuel oil, and natural gas. The carbon emission calculation formula of industrial or construction lands is as follows:

$$C_{T}=\sum W_{i}=\sum w\times U_{i}\times L_{i}\times P_{i}$$

where *C*_*T*_ is the total carbon emission; *W*_*i*_ is the carbon emission of the *i*th energy consumption; *w* is the carbon oxidation rate, which is 100% by default; *U*_*i*_ is the consumption of the *i*th energy; *L*_*i*_ is the average low calorific value of the *i*th energy; and *P*_*i*_ is the emission coefficient of the *i*th energy. The values of *L*_*i*_ and *P*_*i*_ were mainly references from a previous study and the IPCC (2006) [47]. Moreover, the *U*_*i*_ was mainly collected from the China Energy Statistical Yearbook and the Laiwu Statistical Yearbook. The spatial carbon metabolisms of industrial and construction lands were allocated evenly to the pixels of each LULC type based on the value of *C*_*T*_.

### 3.2 Decoupling analysis of carbon metabolism and GDP

The concept of decoupling was put forward by the Organization for Economic Cooperation and Development in 2002 when verifying the relationship between economic development and environmental pollution in various countries [48]. The Tapio decoupling model is the result of further improvement based on the decoupling model. At present, the Tapio decoupling model is a scientific tool used to measure the relationship between the ecological environment and economic development, aiming to measure whether economic development is sustainable [3,11,49,50].

The Tapio decoupling model was also selected in the decoupling analysis of carbon metabolism and GDP in this study. The carbon metabolism and GDP of each unit were extracted by the spatial sum statistics function of ArcGIS based on the raster data of carbon metabolism and GDP. The calculation formula of the decoupling degree between carbon metabolism and GDP is as follows:

$$T_{\left(C,G\right)}=\frac{\Delta C_{t-0}/C_{0}}{\Delta G_{t-0}/G_{0}}=\frac{(C_{t}-C_{0})/C_{0}}{(G_{t}-G_{0})/G_{0}}$$

where *T*_(*C*,*G*)_ is the decoupling degree of carbon metabolism and GDP; Δ*C*_*t*−0_ is the change in the value of carbon metabolism from the base period to the end period; *C*_0_ is the carbon metabolism in the base period; Δ*G*_*t*−0_ is the change in the value of GDP from the base period to the end period; and *G*_0_ is the GDP in the base period.

**Fig 3:**
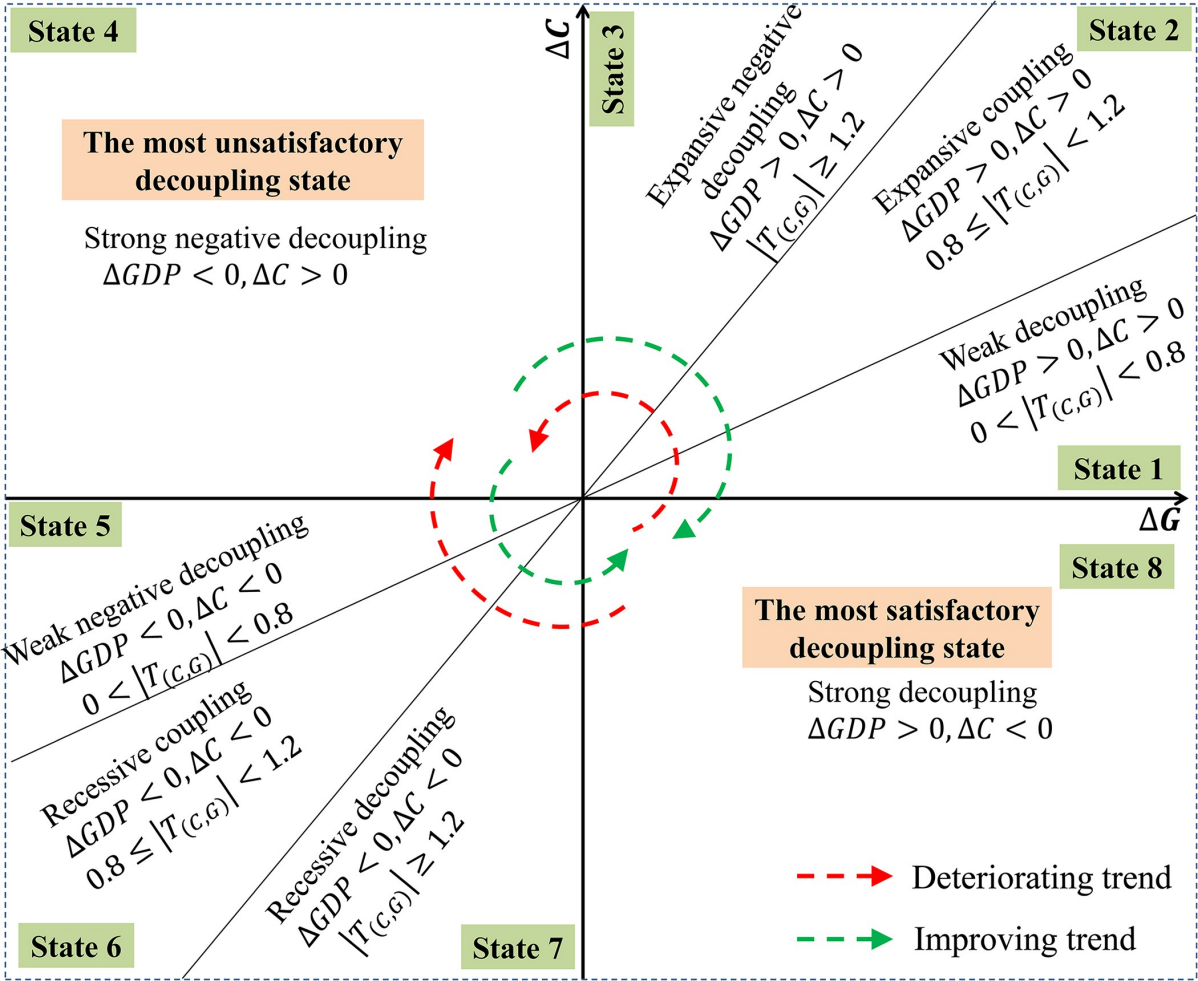
Determination of the decoupling state.

According to the classification standard of Tapio [11,48,51], eight kinds of decoupling states were determined by the values of the decoupling index (Fig 3). Because carbon sequestration is expressed with a negative value in this study, the absolute value of carbon metabolism was used to determine decoupling states. The corresponding interpretation of each state was also expressed from carbon emission and carbon sequestration views as follows:

**State 1 (WD):** The rate of carbon emission or sequestration is less than that of GDP growth.
**State 2 (EC):** The rate of growth of carbon emissions or the rate of decline of carbon sequestration is relatively equal to the rate of growth of GDP.
**State 3 (END):** The rate of growth of carbon emissions or the rate of decline of carbon sequestration is much greater than the rate of growth of GDP.
**State 4 (SND):** Carbon emissions increase while GDP decreases.
**State 5 (WND):** The rate of decline of carbon emissions or the rate of growth of carbon sequestration is largely less than the rate of decline of GDP.
**State 6 (RC):** The rate of decline of carbon emissions or the rate of growth of carbon sequestration is relatively equal to the rate of decline of GDP.
**State 7 (RD):** The rate of decline of carbon emissions or the rate of growth of carbon sequestration is much greater than the rate of decline of GDP.
**State 8 (SD):** carbon emissions decrease while GDP increases.

As shown in Fig 3, the eight kinds of decoupling states were distributed in the quadrant diagram constructed by the coordinate axis of carbon metabolism and GDP. Considering that China, as a developing country, requires further development of its economy, this remains of high importance to China. In the first quadrant of Fig 3, the development trend gradually worsens along the counterclockwise direction from state 1 to 3. In the third quadrant, the development trend gradually improves along the counterclockwise direction from state 5 to 7. In the second quadrant, the development trend is the most unsatisfactory state. In the fourth quadrant, the development trend is the most satisfactory state.

### 3.3 The spatiotemporal pattern analysis of the decoupling degree

The decoupling degrees between carbon metabolism and GDP in 2001–2009 and 2009–2018 in village and town administrative units were separately extracted through the previous steps. Referring to the theory of land change trajectory [52], this was expressed by the “from-to” model of LULC classification for different years. Therefore, the spatiotemporal pattern of the decoupling degree from 2001–2018 was also analyzed by the “from-to” model, such as “EC→END” and”EC→SD” in this study.

Firstly, the spatial patterns of decoupling states of 2001–2009 and 2009–2018 were analyzed separately. The main distribution areas of each decoupling state were presented as maps. Moreover, the proportion of each decoupling state in the village units was determined. The difference in decoupling states between villages and towns was compared.

Secondly, the change of the decoupling state from 2001–2018 was extracted based on the decoupling state of two periods. The proportion of each decoupling state change type in the village units was determined. Then, these change types were grouped into some development trends based on the rules summarized in a previous section. Namely, as shown in Fig 3, the change types along the counterclockwise direction from the fourth to the second quadrant were grouped as the eco-deteriorated economic development (EDED) type. The change types along the counterclockwise direction from the second quadrant to the fourth quadrant were grouped as the eco-improved economic development (EIED) type. Considering the importance of current economic development, the change types from the first to the third quadrant were also grouped as the EDED type.

## 4. Results

### 4.1 The spatiotemporal analysis results of carbon metabolism

**Fig 4:**
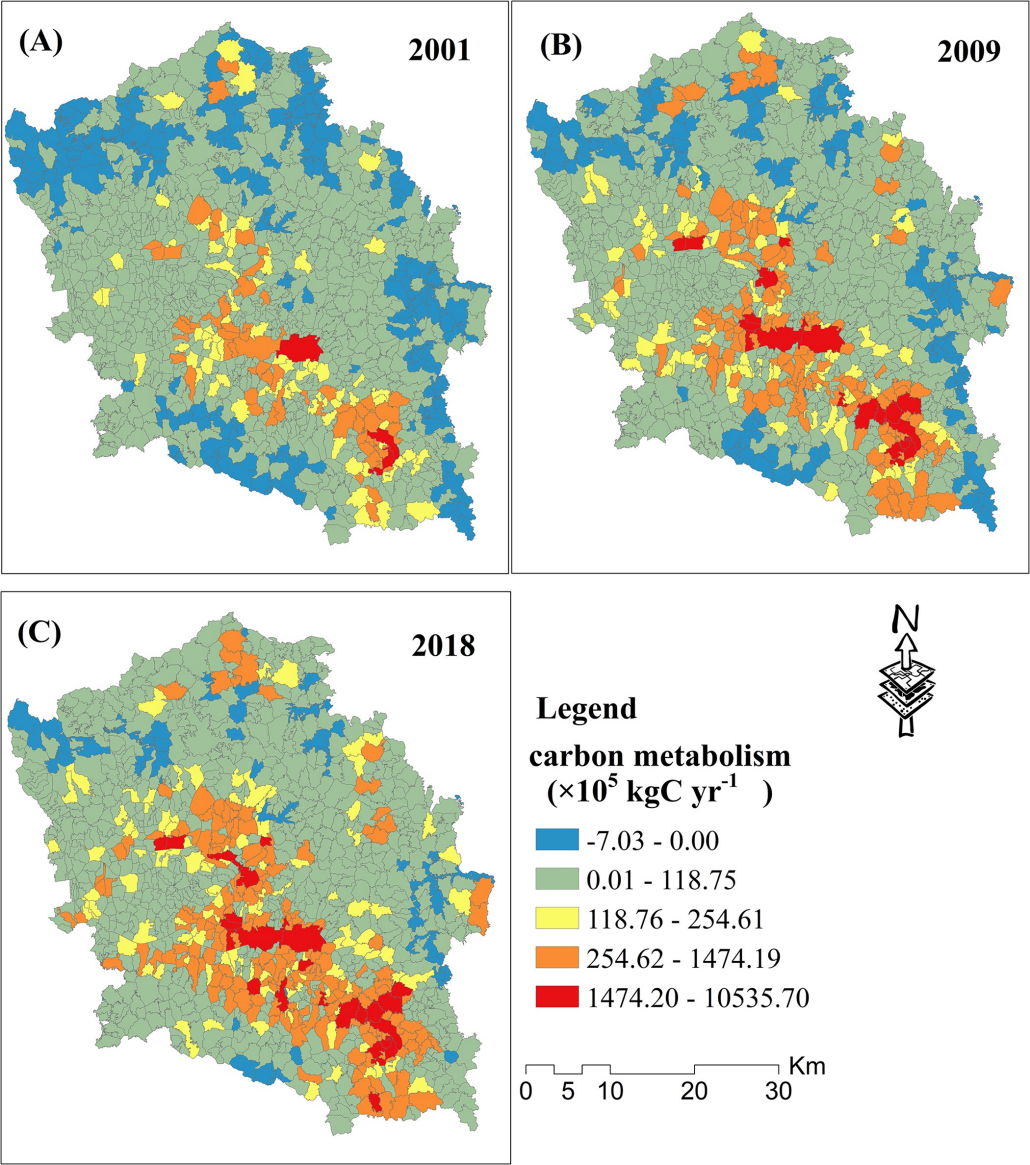
Carbon metabolism for 3 years at the village unit scale.

**Fig 5:**
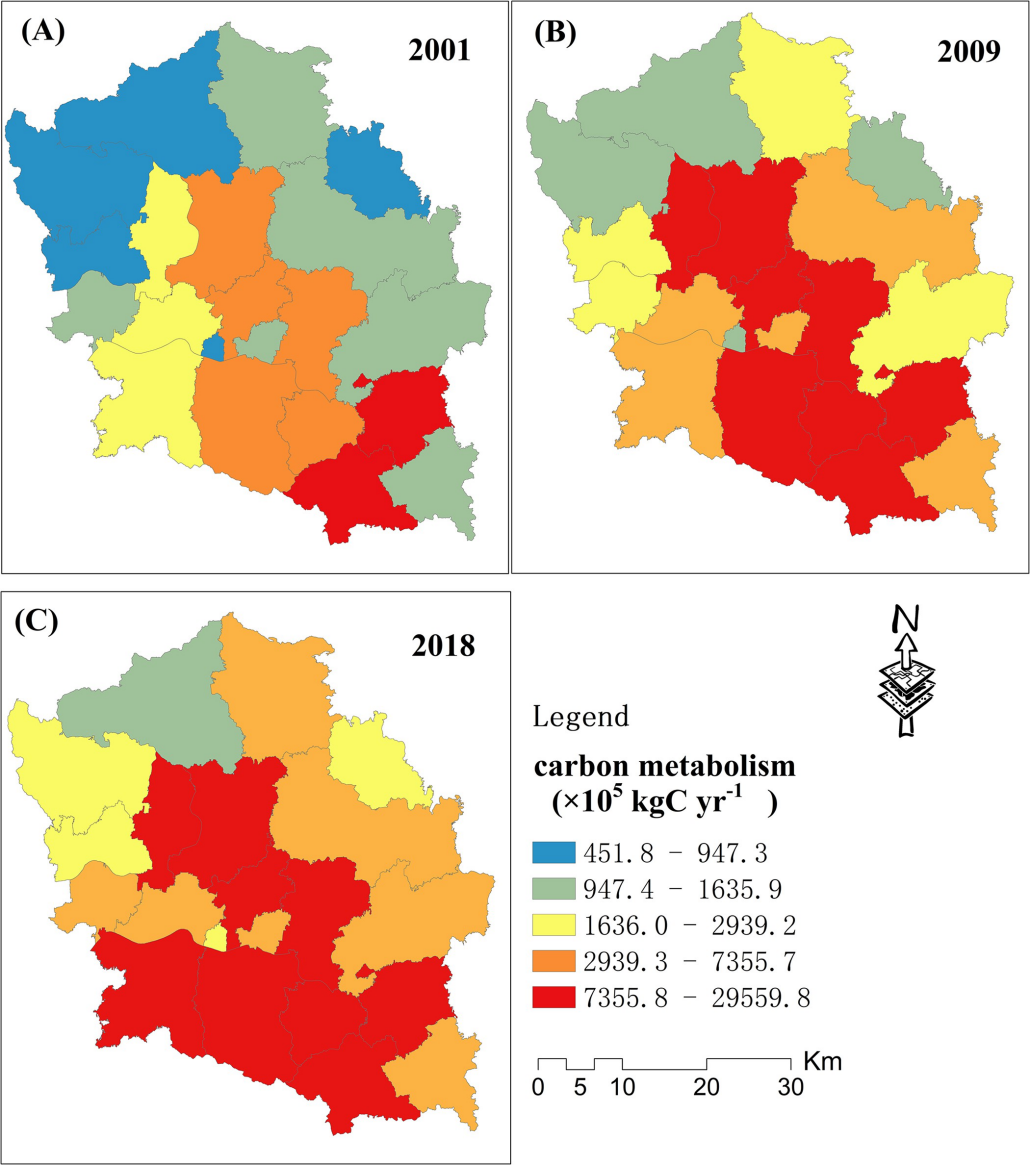
Carbon metabolism for 3 years at the town unit scale.

The carbon metabolism results for village and town administrative units over a 3-year period are shown in Figs 4 and 5. A positive value represents net carbon emissions and a negative value represents net carbon sequestration (Fig 4). The carbon sequestration villages were mainly distributed in the north, southeast, and southwest of the study area. Moreover, the number of carbon sequestration villages showed a significant decrease trend from 2001 to 2018. Most villages presented significant carbon emissions. In addition, the villages with relatively high carbon emissions were mainly distributed in the central and southern parts of the study area. As shown in Fig 5, all of the towns presented significant carbon emissions. The towns with relatively low carbon emissions were mainly distributed in the northern parts, and those with high carbon emissions were mainly distributed in the southern parts, of the study area. Except for a few towns in the north, most presented a significant carbon emission increase trend.

**Fig 6:**
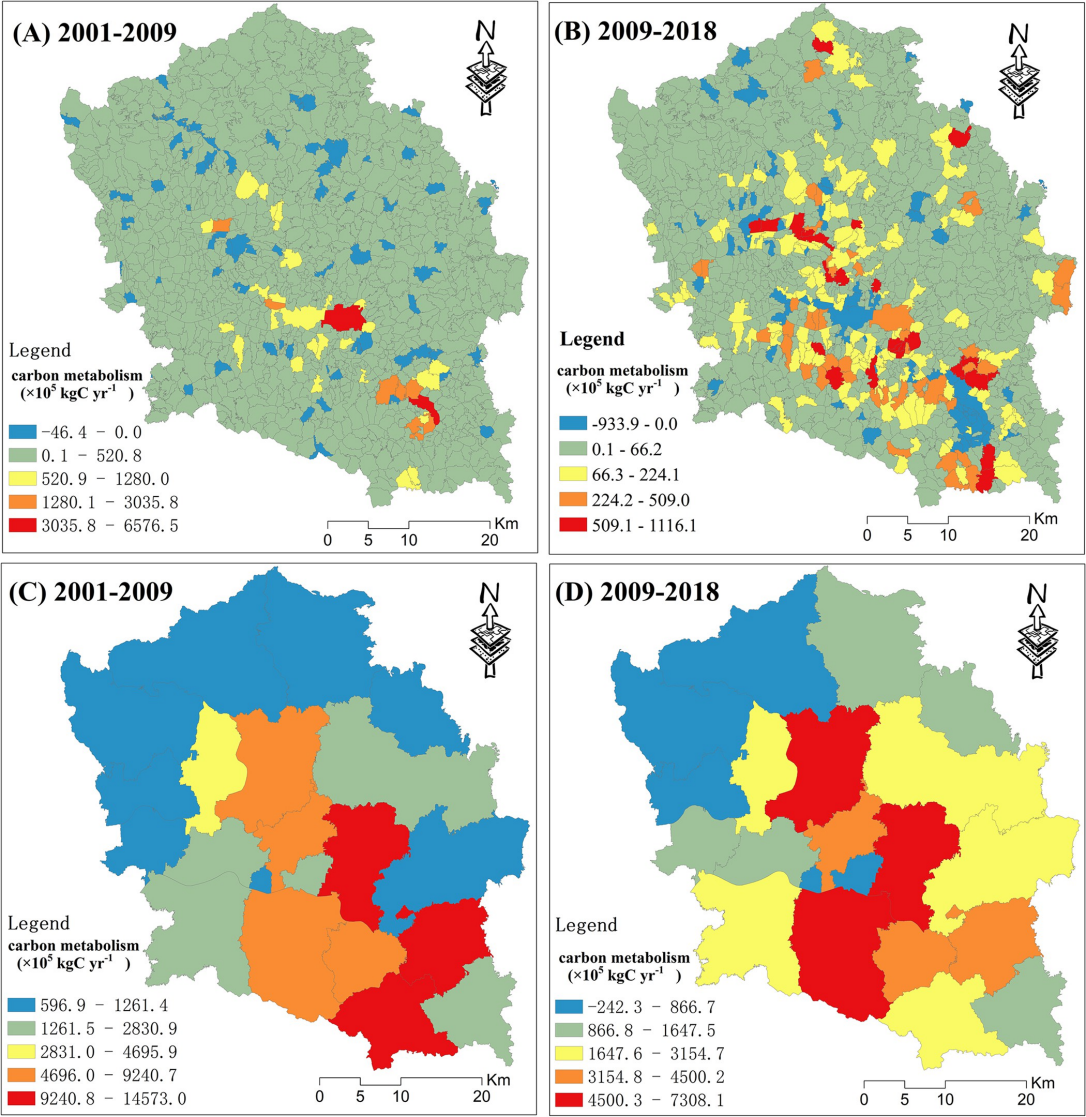
Maps of the carbon metabolism change for two periods at the village and town unit scales.

Maps of the carbon metabolism change over two periods are shown in Fig 6. Most villages present a significant carbon emission growth trend from 2001 to 2009 (Fig 6(A)). A few villages that presented a carbon emission decline trend were scattered and uniformly distributed in the study area. The villages with carbon emission growth exceeding 1,000×10^5^*kgCyr*^−1^ were distributed in the central southern parts of the study area. As shown in Fig 6(B), most villages also presented a significant carbon emission growth trend from 2009 to 2018. However, the amount of growth was significantly lower than that from 2001–2009. A few villages that presented a carbon emission decline trend from 2009–2018 were mainly clustered in the southern and central parts of the study area. All towns presented a significant carbon emission growth trend from 2001–2009 (Fig 6(C)). Moreover, carbon emissions growth showed a spatial distribution trend of high in the south and low in the north. In addition, the amount of growth of all towns was significantly lower than that from 2001–2009. A few towns in the northwest and central parts of the study area presented a carbon emission declining trend from 2009–2018 (Fig 6(D)).

### 4.2 Spatiotemporal analysis results of the decoupling state

**Fig 7:**
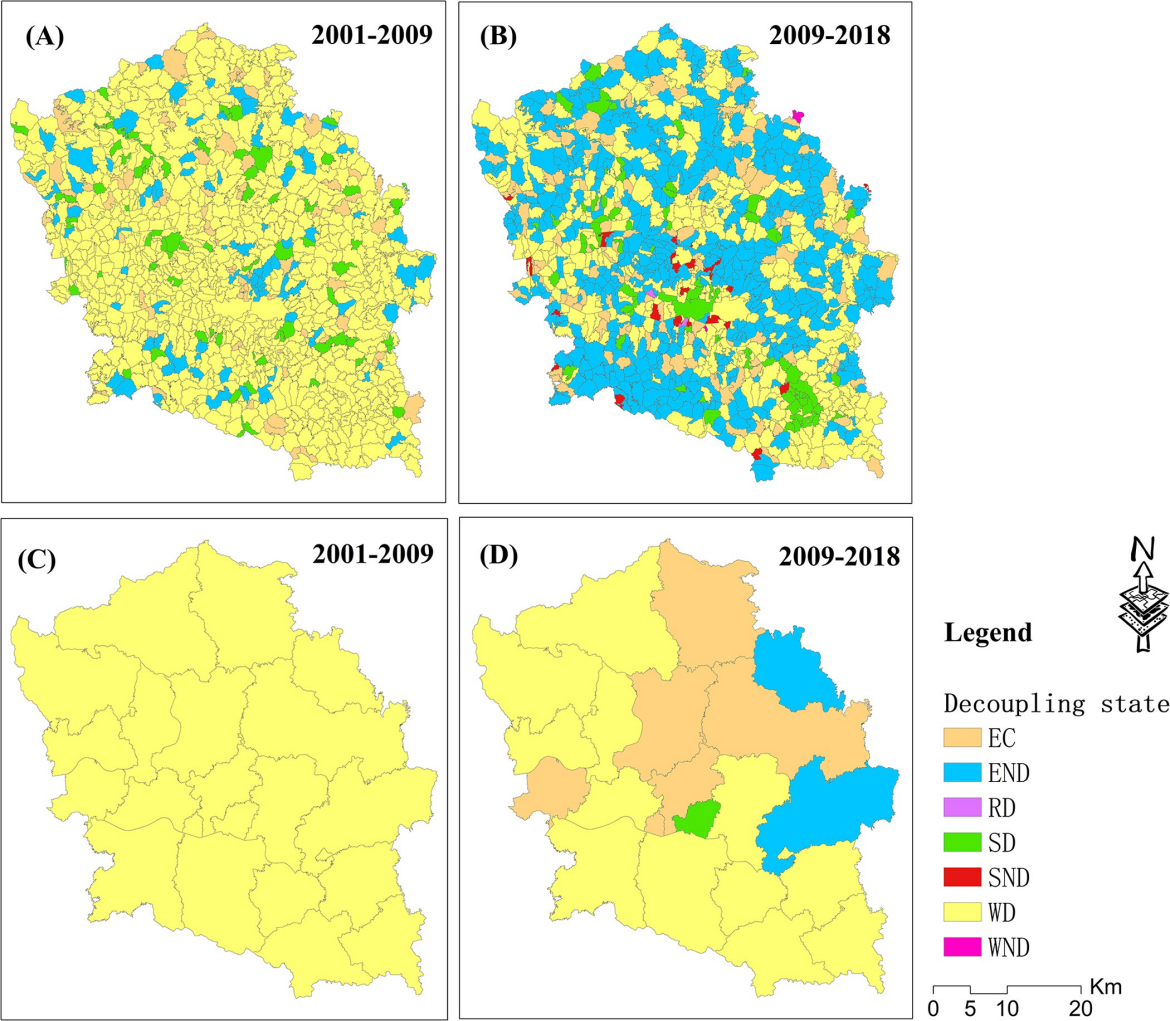
Decoupling state between the carbon metabolism and GDP across two periods at the village and town unit scales. EC, Expansive coupling; END, Expansive negative decoupling; RD, Recessive coupling; SD, Strong decoupling; SND, Strong negative decoupling; WD, Weak decoupling; WND, Weak negative decoupling.

**Fig 8:**
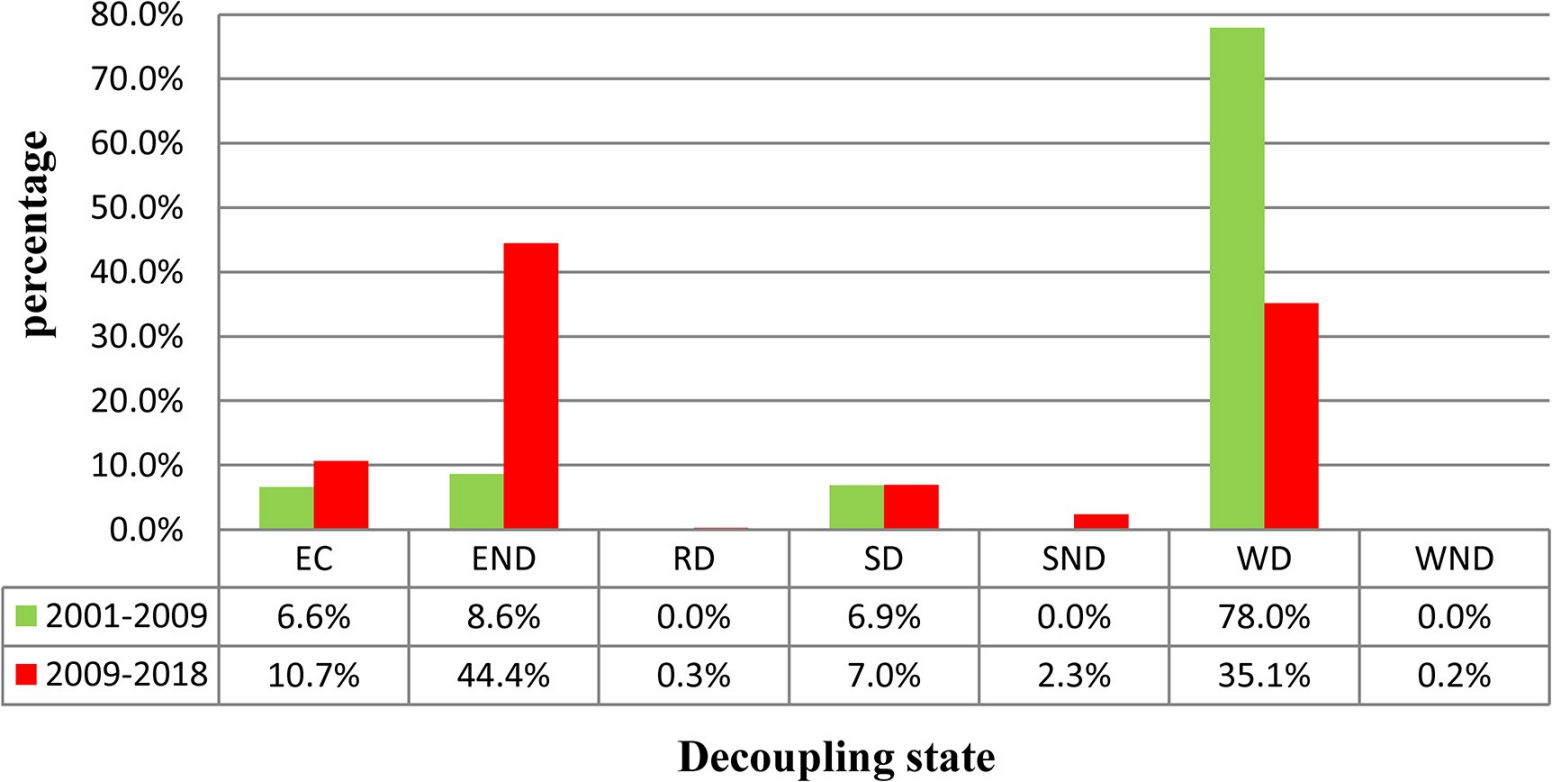
The proportion of villages of each decoupling state for carbon metabolism and GDP across two time periods.

The spatial distribution of the decoupling state between the carbon metabolism and GDP in two periods is presented in Fig 7. Moreover, the proportion of villages of each decoupling state in the two periods is shown in Fig 8. From 2001–2009, the state of all towns was WD, meaning that economic development mostly drove carbon emissions. Approximately 6.6% of villages had the state EC, 8.6% had the state END, 78% had the state WD, and 6.9% had the state SD, which was the most ideal development state. Moreover, the spatial pattern of the SD villages did not have obvious agglomeration characteristics. This means that most villages showed a relationship between their economic development and carbon emissions. Approximately 78% of villages had the same decoupling status as the towns, 15.2% had a worse decoupling status than the towns, and 6.9% had a better decoupling status than the towns (Fig 10).

As shown in Figs 7 and 8, from 2009–2018, the decoupling state of six towns changed from WD to EC, that of two towns changed from WD to EC, and that of one town changed from WD to SD. The villages of EC occupied about 10.7% and generally presented a uniform distribution pattern. The villages of END occupied about 44.4% and had obvious agglomeration distribution characteristics in the southwest, central, and northern regions of the study area. The villages of SD occupied about 7% and did not show obvious changes compared to 2001–2009. However, the spatial pattern of SD presented obvious agglomeration characteristics, with clustering mainly in the central and southern parts of the study area. The villages of SND occupied about 2.3% and were mainly distributed in the central and southern regions of the study area. The proportion of villages with the decoupling state WD decreased from 78% to 35.1%.

**Fig 9:**
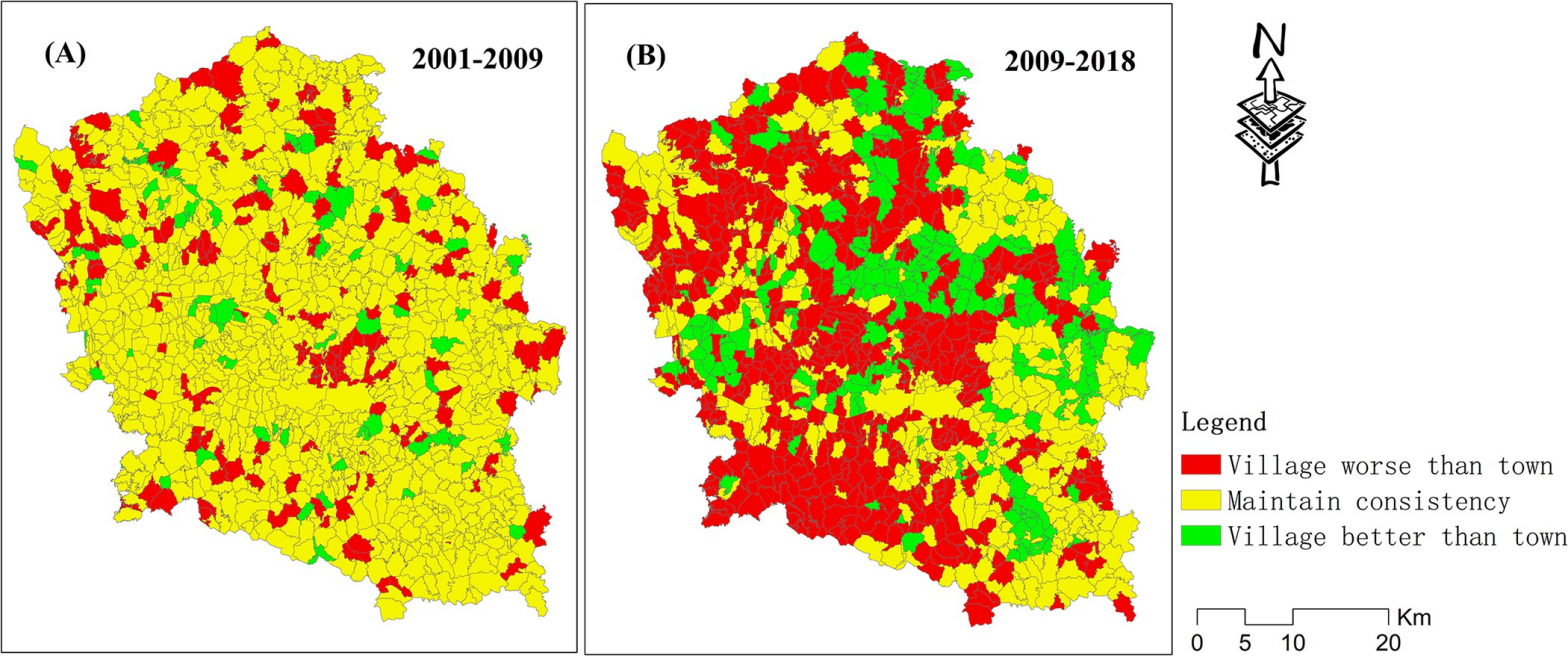
Analysis results of the decoupling statuses of villages and towns.

**Fig 10:**
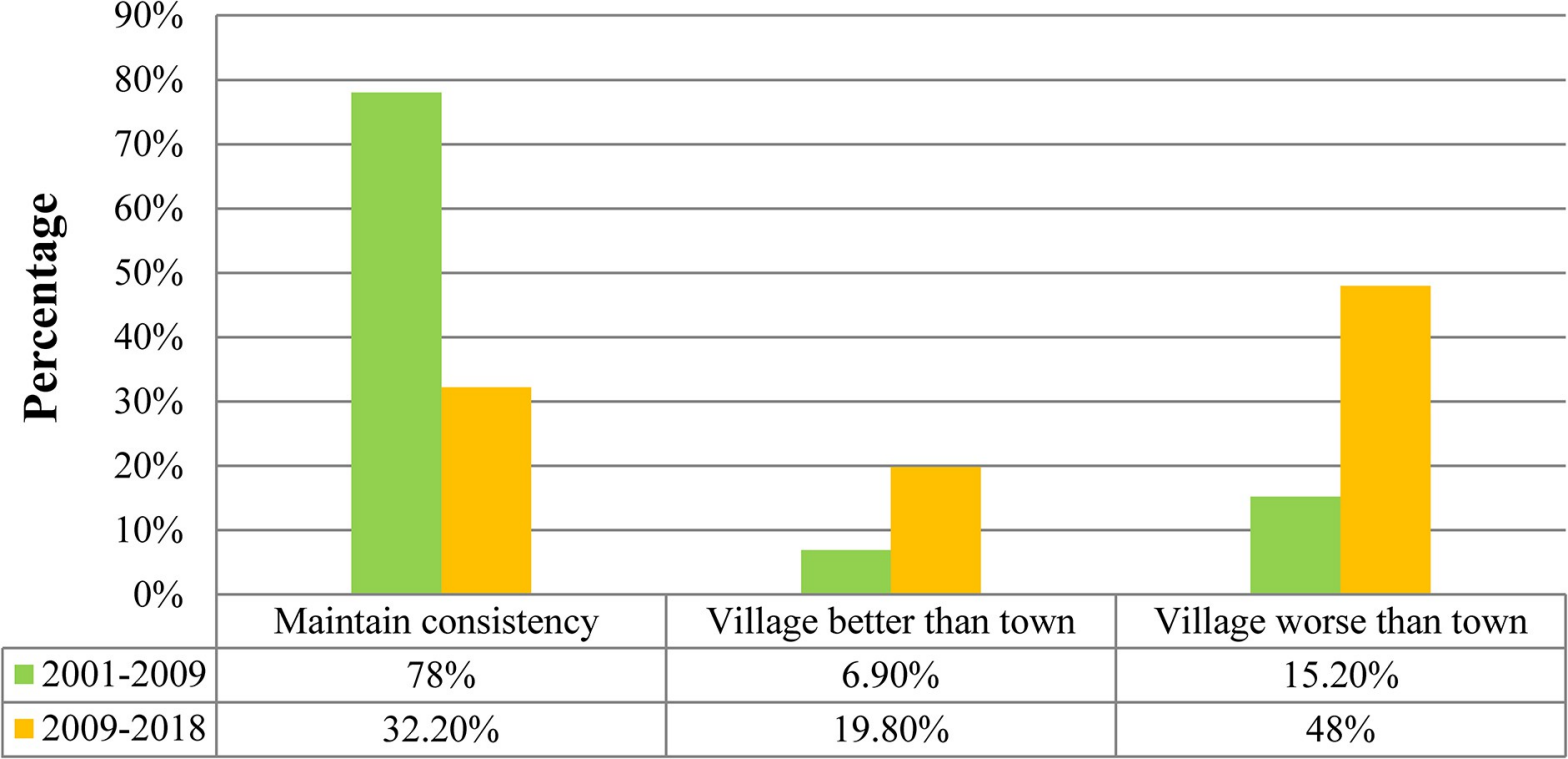
Decoupling status differences between villages and towns across two time periods.

This study analyzed the difference in decoupling state between villages and towns by spatially stacking the decoupling results, as shown in Fig 7, across two periods. The comparative analysis results are shown in Figs 9 and 10. From 2001–2009, the decoupling status of approximately 78% of villages was consistent with that of towns. Approximately 6.9% of villages had a better decoupling status than the towns, and approximately 15.2% of villages had a worse decoupling status than the towns. From 2009–2018, the decoupling status of approximately 32.2% of villages was consistent with that of towns. Approximately 19.8% of villages had a better decoupling status than the towns, and approximately 48% of villages had a worse decoupling status than the towns (Fig 10).

### 4.3 Evolution types of the decoupling state from 2001–2018

**Fig 11:**
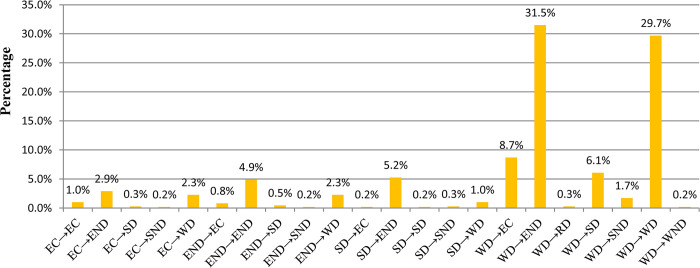
Proportion of villages of each decoupling state change type across two time periods.

Based on the decoupling state types of villages across the two time periods, 22 change types of the decoupling state were extracted from 2001–2018 (Fig 11). The change types of WD→END and WD→WD separately occupied approximately 31.5% and 29.7% of villages. The change types of EC→END, EC→WD, END→END, END→WD, SD→END, WD→EC, WD→SD, and WD→SND together occupied about 34.1% of villages, and other change types occupied about 4.7%.

Referring to the definition rules of each decoupling state (Fig 3), the decoupling state change types were grouped into four evolution types (Table 2). The stable eco-consumption economy development (SECED) type contained the unchanged decoupling states that maintained the traditional development trend of simultaneous GDP and carbon metabolism growth. The EDED type contained the decoupling state change types from strong to weak decoupling, or from a weak to a worse decoupling state. The EIED type contained the decoupling state change types from weak to strong decoupling, or from unsatisfactory to more satisfactory decoupling. The stable eco-friendly economic development type (SEFED) contained the unchanged decoupling state that maintained the most ideal development trend.

**Table 2:** Decoupling state evolution types and their corresponding decoupling state change types.

Evolution types	Decoupling state change types
Stable eco-consumption economy development type (SECED)	EC→EC, END→END, WD→WD
Eco-deteriorated economic development type (EDED)	EC→END, EC→SND, END→SND, SD→EC, SD→END, SD→SND, SD→WD, WD→EC, WD→END, WD→RD, WD→SND, WD→WND
Eco-improved economic development type (EIED)	EC→SD, EC→WD, END→EC, END→SD, END→WD, WD→SD
Stable eco-friendly economic development (SEFED)	SD→SD

**Fig 12:**
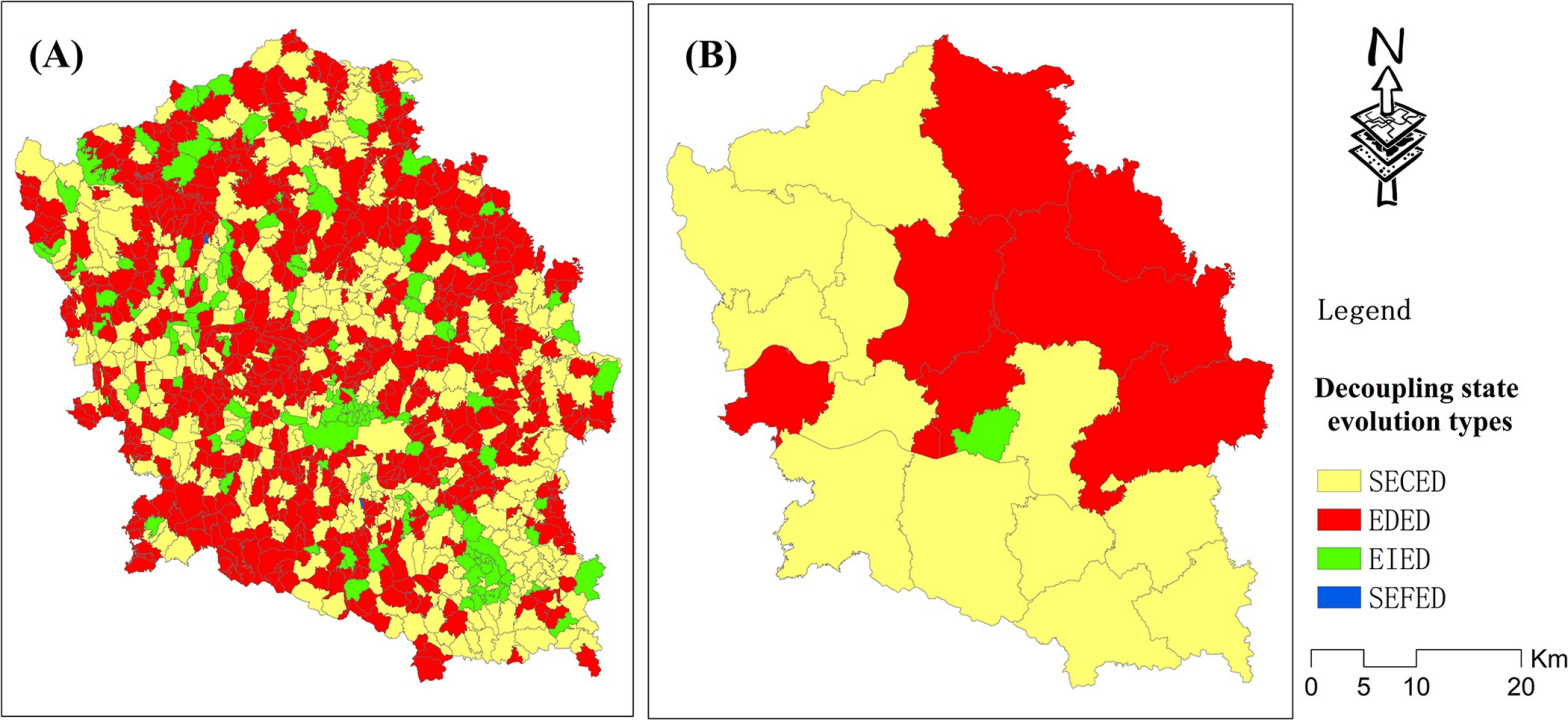
Maps of the decoupling state evolution types from 2001–2018 in the village and town unit scales.

**Fig 13:**
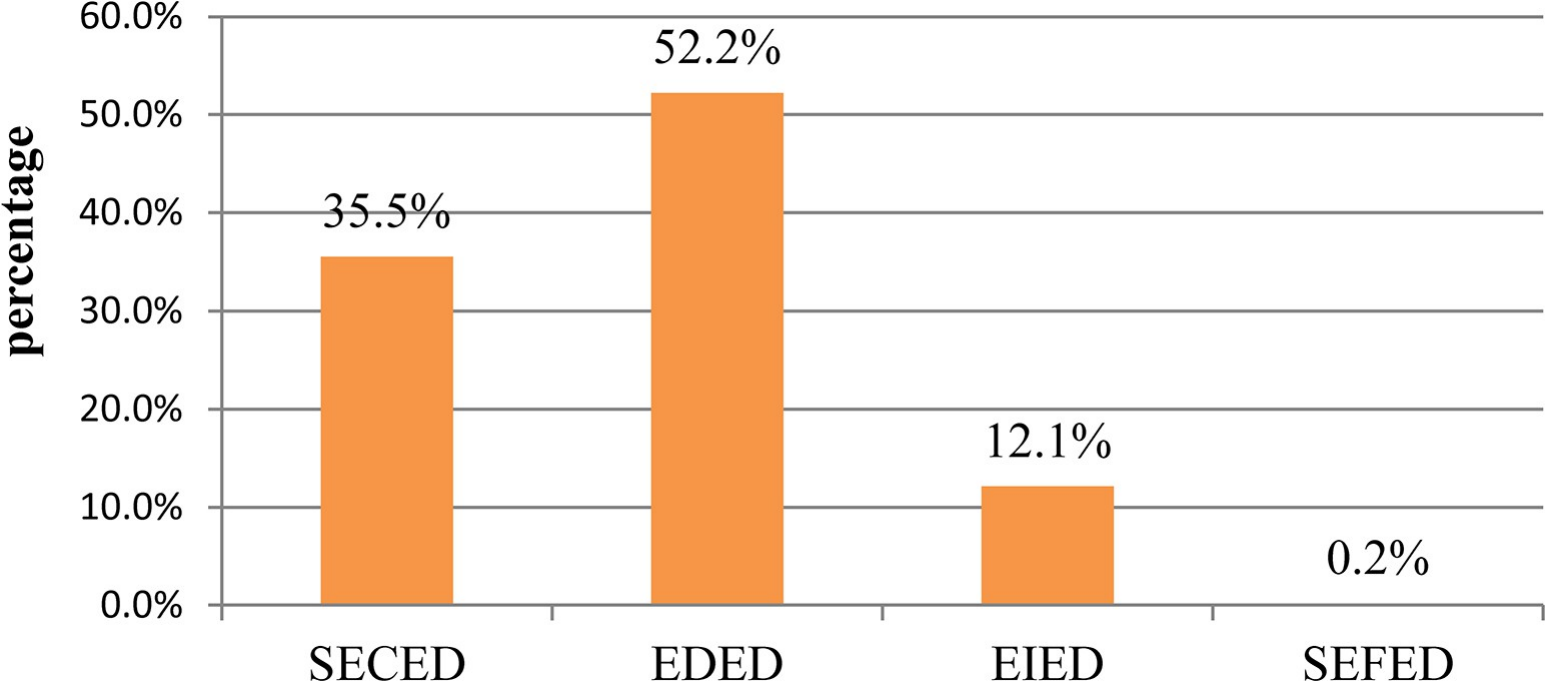
Proportion of villages for each decoupling state evolution type.

The spatial distribution pattern of these evolution types in village and town units is displayed in Fig 12, which shows that 12 towns had the evolution type of SECED and were mainly distributed in the western and southern parts of the study area. Seven towns had the evolution type EDED and were mainly distributed in the northeastern part of the study area. One town had the evolution type EIED and was distributed in the center of the study area. As shown in Figs 12 and 13, approximately 35.5% of villages had the evolution type of SECED and presented a spatial uniform agglomeration distribution pattern. Approximately 52.2% of villages had the evolution type of EDED and also presented a spatial uniform agglomeration distribution pattern. Approximately 12.1% of villages had the evolution type of EIED and were mainly cluster-distributed in the central and southern parts and scatter-distributed in the northern part of the study area.

## 5. Discussion

This study assessed the decoupling state between carbon metabolism and economic development of the specified study area across two time periods and compared the development trends between different administrative units. The purpose of this study was to provide a method of rural development analysis on the one hand, and on the other hand reveal the economic and environmental development status of villages and towns in Laiwu, which is a representative area to investigate in eastern China, in the context of the rural revitalization policy.

According to the results, the growth of carbon metabolism in the study area from 2001–2009 was significantly higher than that from 2009–2018. This is because China mainly focused on economic development from 2001–2009 [53], and the pillar industry of the study area is the steel industry, which is mainly supported by coal consumption. From 2009–2018, the government has gradually increased the importance of environmental protection [54]. The steel industry of Laiwu has mostly moved to the coastal regions. At the same time, most sectors in the study area have replaced coal with natural gas as their main energy source. The regions with low carbon metabolism are mainly distributed in the north of the study area, because most of the mountains and forests in the study area are located in the north. The regions with a high carbon metabolism are mainly distributed in the central and southern parts of the study area, as they are mostly urbanized and contain the towns and industries.

As one common method for analyzing the consistency between economic development and ecological trends, the decoupling analysis results of the two time periods mostly presented different states in two administrative units. Since China’s accession to the WTO in 2001, 2001–2009 was a period of rapid economic development in China. Due to the low economic volume before 2001, the economic growth rate from 2001–2009 was relatively high. Therefore, the decoupling state of all towns and most villages was WD from 2001–2009.

From 2009–2018, most villages and towns transformed from WD to EC or END. This means that although most regions still maintained the growth trend of carbon metabolism and economic development from 2009–2018, the growth rate of GDP was mostly lower than the growth rate of carbon metabolism. There are two possible reasons for this trend. Firstly, in 2009, the capital had experienced around 10 years of development. Therefore, it is difficult to achieve a relatively high economic growth rate from 2009–2018 based on the large economic base. Secondly, the early economic development of China was mainly driven by heavy industry [55]. With the emergence of some environmental problems, the government realized the importance of environmental protection and industrial upgrading [56]. Some measures have been carried out, such as industrial merging and relocating, the promotion of clean energy, and the delineation of ecological protection red lines [57,58]. Under these influences, although most regions have maintained the growth trend of carbon metabolism and economic development from 2009–2018, the increased amount is still significantly less than that from 2001–2009.

Through analysis, it was found that the decoupling status between the villages and towns had a high degree of consistency from 2001–2009 and of inconsistency from 2009–2018. From 2001–2009, the decoupling status of about 78% of villages was consistent with that of the towns. Moreover, from 2009–2018, the proportion of consistency reduced to 32.2%, and the decoupling state of about 48% of villages was weaker than that of the towns. This to some extent indicated that the development trend gradually tended towards spatial imbalance in the study area. Most villages can no longer keep up with the development pace of towns. The reason for this trend is that, according to the theory of agglomeration economy [59], with economic development, spatial imbalance of the economy will inevitably occur. Rural productivity generally flows to cities and towns because the income from engaging in agricultural production is lower than that from working in cities. Therefore, large amounts of mobile equipment and chemical fertilizers are applied in agricultural production. These all lead to an increase in carbon emissions. Therefore, the development of ecological agriculture industrialization is an effective method for future rural development.

In view of the overall development trend of the decoupling state from 2001–2018, approximately 52.2% of villages were the EDED type. However, this does not necessarily mean that it is a negative phenomenon. As previously mentioned, China has been increasing measures to achieve industrial upgrading and environmental protection since 2009. During this period, the economic growth has inevitably slowed. In addition, the decoupling state was extracted from the comparison values between GDP growth and carbon growth. Therefore, in the short term, the growth rate of GDP is less than that of carbon emissions. If industrial transformation and upgrading are successful, it is possible that the decoupling state will enter a satisfactory trend. The EIED type is mainly distributed in city centers that are mainly agglomerated in the central and southern parts of the study area. Previously, heavy industry was mainly distributed around cities. However, in recent years, these heavy industries have been controlled or relocated to other places. Therefore, carbon emissions in these areas have presented significant decreases.

Although this study successfully revealed the spatial characteristics and spatiotemporal evolution patterns of decoupling states between economic development and carbon metabolism, as well as their differences in different administrative units, there remain some limitations in this study. Firstly, to extract the carbon metabolisms of the industrial and construction lands at a small scale, the total carbon metabolism values calculated by the fossil energy consumption statistics of each city were distributed evenly to each pixel of the land type. Within industrial land, different industries exist with different carbon metabolic intensities. Moreover, within construction land, different energy use intensities exist that cause different carbon metabolic intensities. Therefore, the average spatial allocation can lead to more or less carbon metabolism evaluation errors. We will aim to solve this problem in future research by considering more detailed spatial facility data. Secondly, here, only one case study was selected to represent the development model of towns in eastern China. Considering the unbalanced development of eastern, central, and western China, it is necessary to select more typical cases from other regions in future. Finally, the main aim of this study was to reveal the spatial characteristics and spatiotemporal evolution patterns of decoupling states between economic development and carbon metabolism. However, there is currently a lack of research available on their influence mechanisms. Therefore, in future research, some impact factors will be selected based on this study to analyze the mechanism of these spatiotemporal evolution patterns.

## 6. Conclusion

This study used the LULC-based carbon metabolism evaluation method to extract the carbon metabolisms of the case area from 2001–2018. Moreover, the administrative boundary of the villages and towns was used to extract the carbon metabolisms in these administrative units using the spatial statistical analysis method. Then, the spatial characteristics and spatiotemporal evolution patterns of decoupling states between the carbon metabolism and economic development were analyzed based on two time periods of 2001–2009 and 2009–2018.

This study showed that the growth rate of carbon metabolism from 2001–2009 was significantly higher than that from 2009–2018. The regions with significant increases in carbon emissions were mainly distributed in the central and southern parts of the study area. The spatial heterogeneity of the decoupling states between economic development and carbon metabolism from 2009–2018 was significantly stronger than that from 2001–2009 in two units. From 2009–2018, about 50% of villages and towns transformed from WD to EC, END, and SD. The decoupling state of END agglomerated in most regions of the study area, and SD was mainly distributed in the regions around city centers at the central and southern parts of the case area.

From 2001–2018, the development trend gradually tended towards spatial imbalance. The decoupling status between villages and towns had a high degree of consistency from 2001–2009 and of inconsistency from 2009–2018. From 2001 to 2009, the decoupling state of about 78% of villages was consistent with that of the towns. Moreover, from 2009–2018, the proportion of consistency reduced to 32.2%, and the decoupling state of approximately 48% of villages was weaker than that of the towns.

From 2001–2018, according to the reclassification results of different decoupling state change types, it could be concluded that approximately 52.2% of villages had a decoupling state evolution type of EDED, which is an unsatisfactory development trend in a short time. Moreover, the EDED type was mainly distributed in rural areas. In addition approximately 12.1% of villages had a decoupling state evolution type of EIED, which is a satisfactory development trend. Finally, the EIED type was mainly distributed around city centers.

Based on this study, the following aspects should be focused on for future development: Firstly, industrial upgrading should be undertaken as a matter of urgency to reduce the dependence of economic development on heavy industry in order to achieve complete decoupling between economic development and carbon emissions. Secondly, to promote balanced urban-rural development, the industrialization of ecological agriculture could serve as a development path.

## Supporting information

S1 File (ZIP)
