# Supplementary figures and images for: Analyzing the spatiotemporal pattern of the decoupling degree between carbon metabolism and economic development in village and town units
Source: PLoS One. 2024 Apr 18;19(4):e0296787. doi: 10.1371/journal.pone.0296787 (PMC11025739; doi:10.1371/journal.pone.0296787)

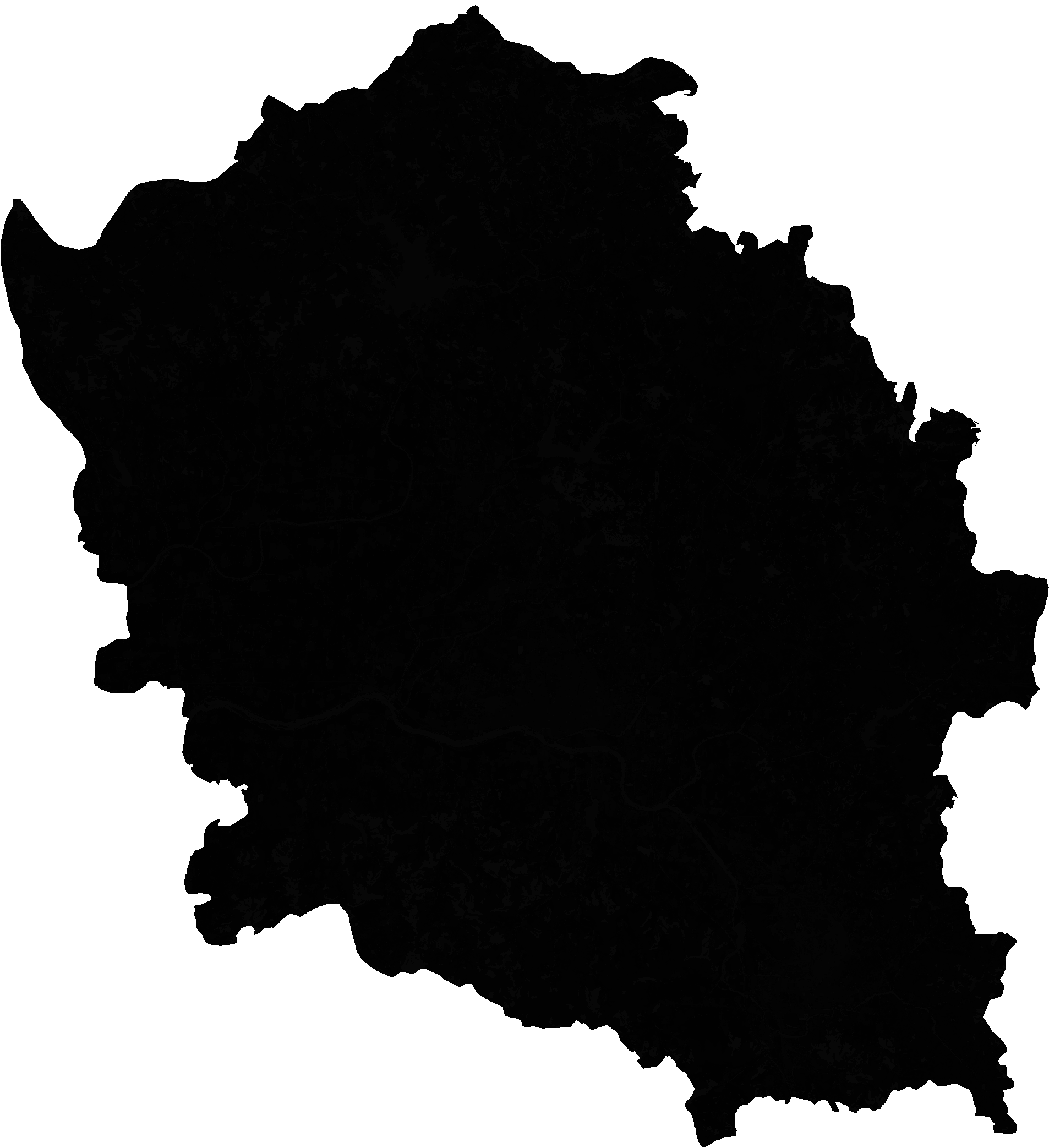

Supplement: S1 File — (ZIP) [file pone.0296787.s001.zip › Supporting Information files/LULC-2001.tif]

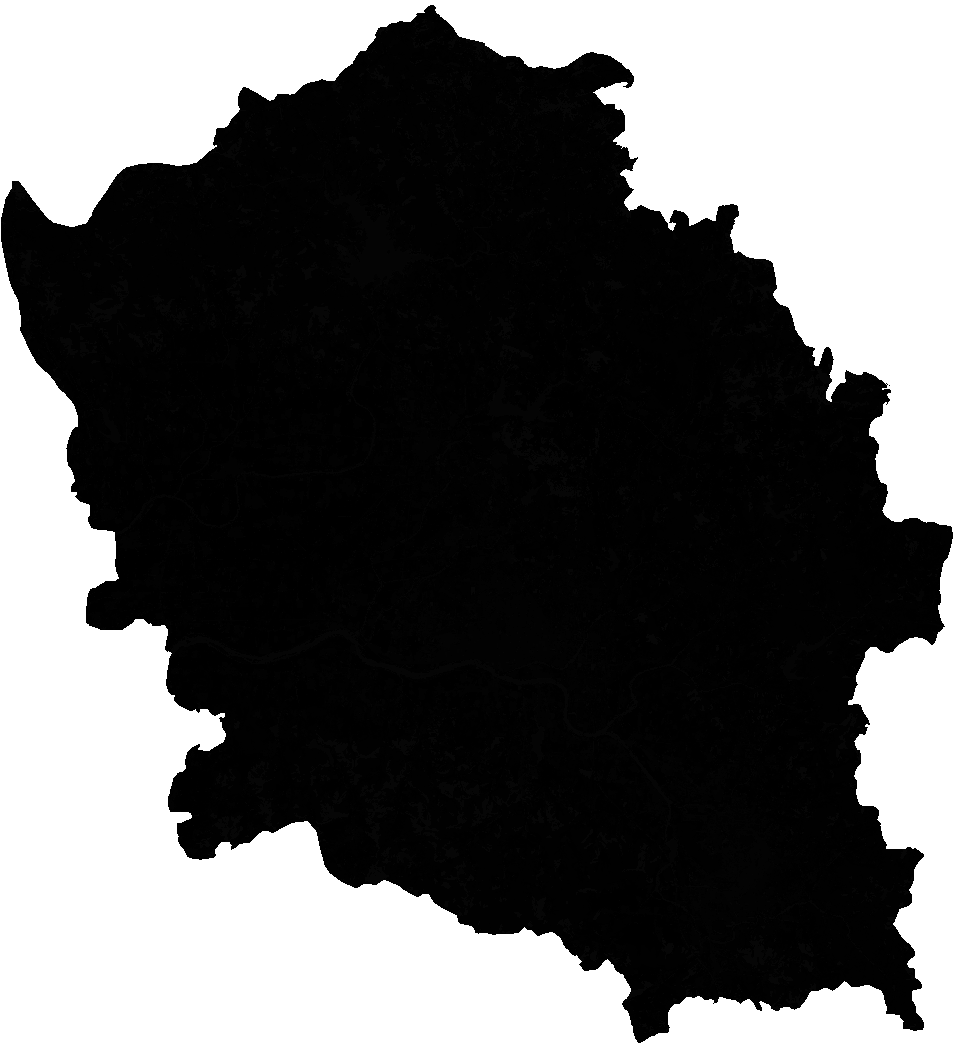

Supplement: S1 File — (ZIP) [file pone.0296787.s001.zip › Supporting Information files/LULC-2001.tif.ovr]

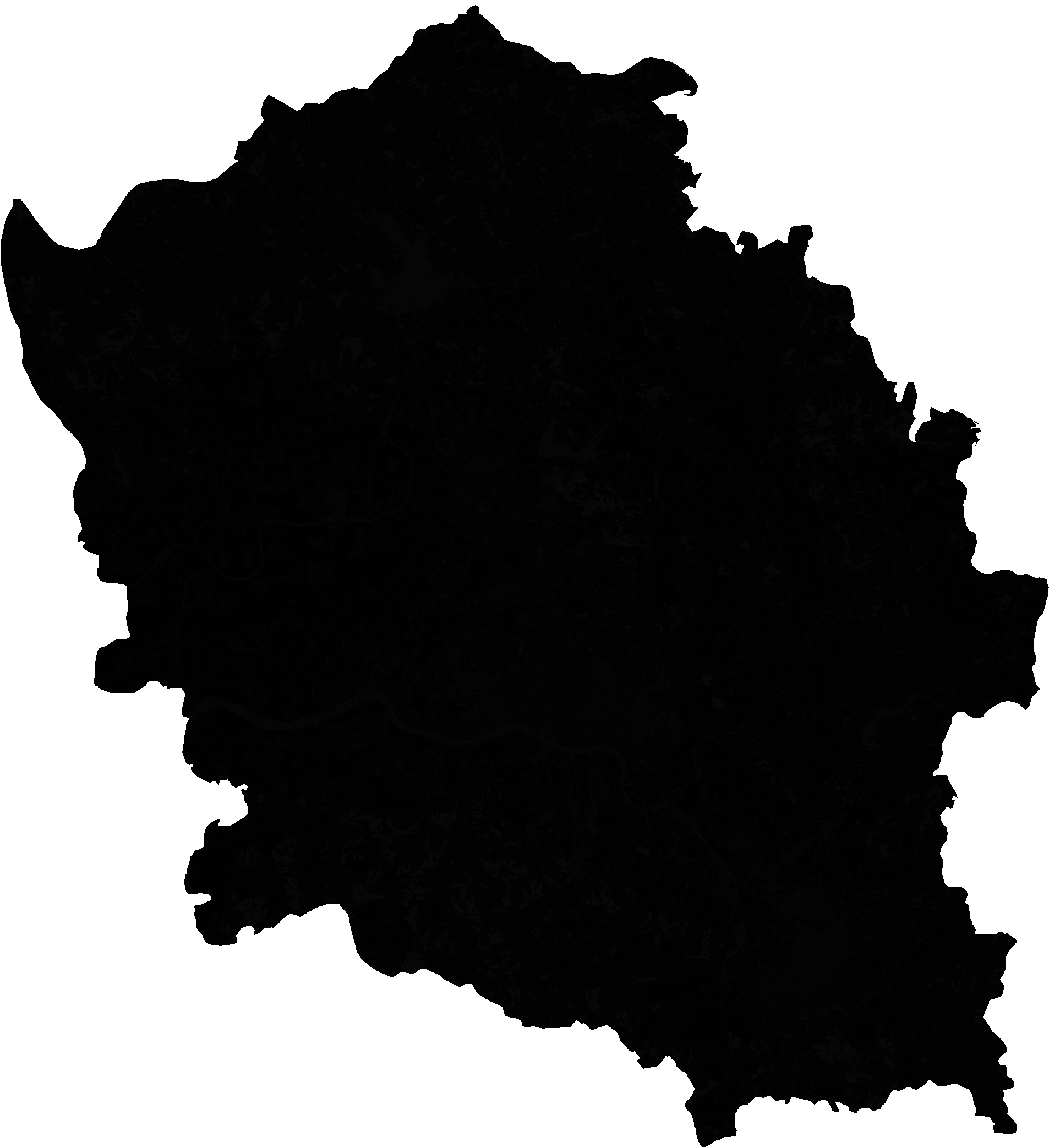

Supplement: S1 File — (ZIP) [file pone.0296787.s001.zip › Supporting Information files/LULC-2009.tif]

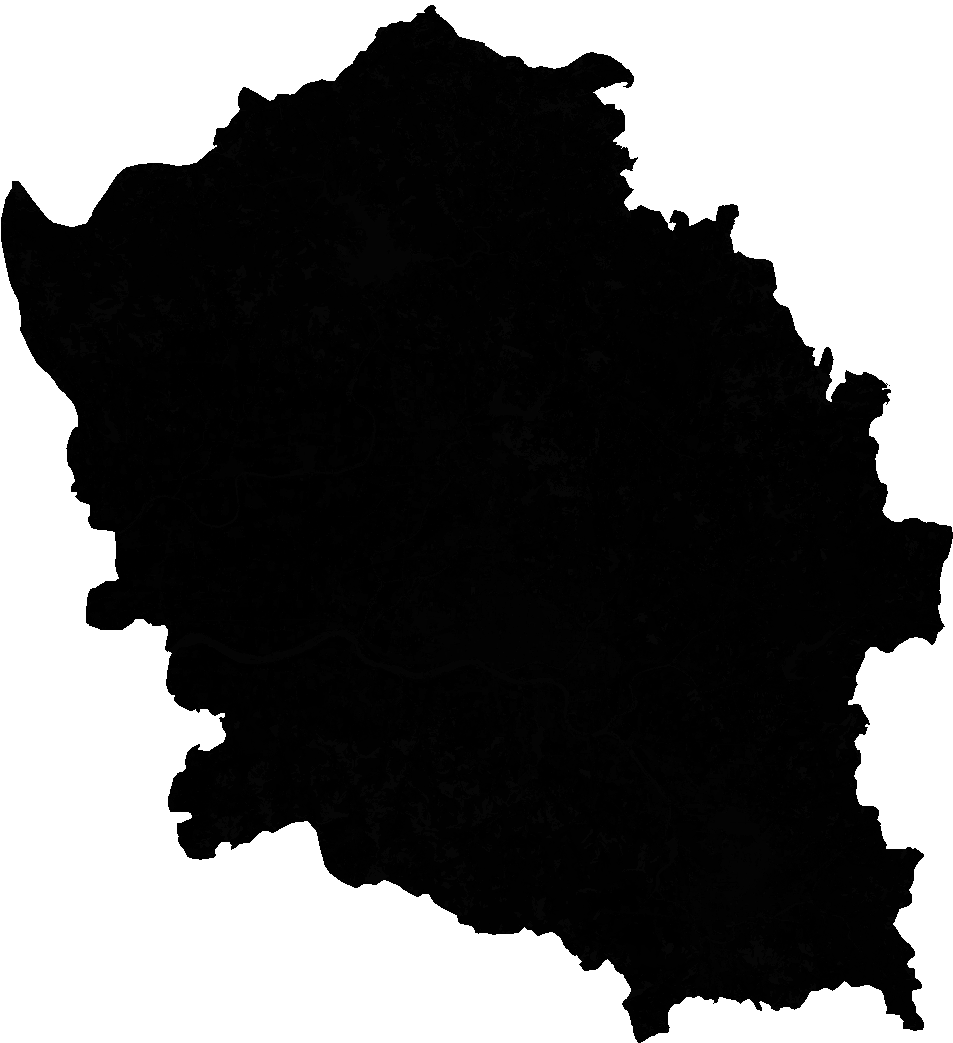

Supplement: S1 File — (ZIP) [file pone.0296787.s001.zip › Supporting Information files/LULC-2009.tif.ovr]

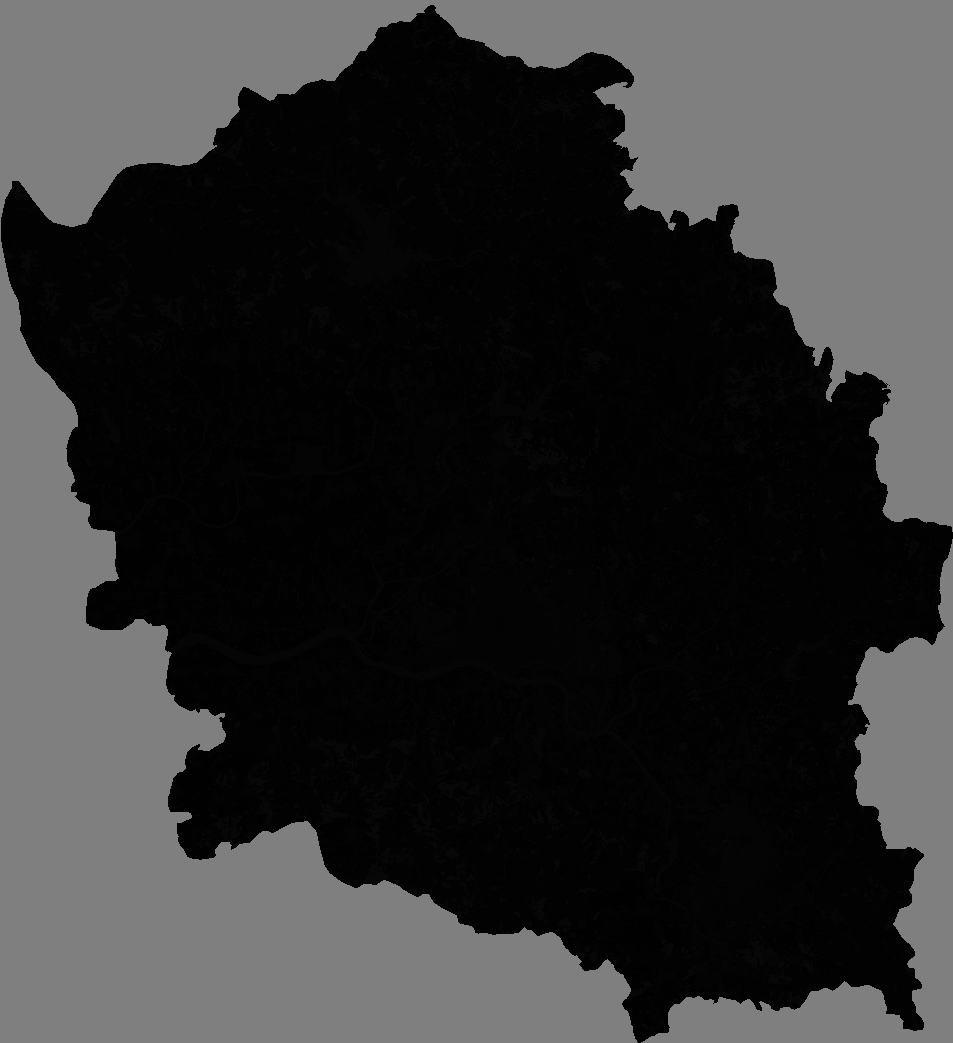

Supplement: S1 File — (ZIP) [file pone.0296787.s001.zip › Supporting Information files/LULC-2018.tif.ovr]
